# The Management of Menopause in Women with Philadelphia-Negative Myeloproliferative Neoplasms: Clinical Challenges and Therapeutic Considerations

**DOI:** 10.3390/cancers18050728

**Published:** 2026-02-24

**Authors:** Claire Woodley, Priya Sriskandarajah

**Affiliations:** Department of Haematology, Guy’s Hospital, London SE1 9RT, UK; clairewoodley@nhs.net

**Keywords:** menopause, myeloproliferative neoplasms, hormone replacement therapy

## Abstract

Philadelphia-Negative Myeloproliferative neoplasms (MPNs) are chronic clonal hematopoietic malignancies characterized by dysregulated myeloid proliferation, chronic inflammation, and an increased risk of thrombotic and hemorrhagic complications. In addition to disease-related morbidity, MPNs are associated with a substantial symptom burden that significantly impacts quality of life. Menopause is accompanied by hormonal changes that can produce symptoms overlapping with those of MPNs, complicating clinical assessment and management in affected women. This challenge is further compounded by the lack of disease-specific guidance on menopause management in women with MPNs and ongoing concerns regarding the thrombotic risk associated with hormone replacement therapy (HRT). This narrative review summarizes the current evidence on menopause in women with Philadelphia chromosome–negative MPNs, with particular focus on the safety and role of HRT, non-hormonal therapeutic alternatives, and supportive care strategies. We also propose a pragmatic clinical algorithm to support individualized menopause management in this high-risk population.

## Simple Summary

Philadelphia-Negative Myeloproliferative neoplasms (MPNs) are chronic blood cancers that can be associated with significant symptoms. These can impact patients’ quality of life and thus require close management. In women, particularly those going through menopause, the symptom burden can increase. However, due to the crossover of symptoms between menopause and MPN, it can be difficult to delineate the two conditions in these patients. This is compounded by the lack of guidance around the use of hormone replacement therapy in MPN patients due to the potential risk of thrombosis. Our review aims to assess evidence to date around menopause and MPN and highlight potential therapy strategies to consider in these patients.

## 1. Introduction

Myeloproliferative neoplasms (MPNs) are a heterogeneous group of clonal hematopoietic stem cell disorders characterized by excessive production of mature myeloid blood cells. The classical Philadelphia chromosome-negative MPNs include polycythemia vera (PV), essential thrombocythemia (ET), and primary myelofibrosis (PMF), with disease pathogenesis most commonly driven by acquired mutations in *JAK2*, *CALR*, and *MPL* genes [1]. These disorders are associated with chronic inflammation, splenomegaly, progressive marrow dysfunction and elevated risks of arterial and venous thrombosis [1,2].

Sex and gender have emerged as central determinants of disease biology, clinical phenotype, and outcomes in cancers [3]. Furthermore, recent data have identified sex-dependent differences at the level of hematopoietic stem cells, impacting hemopoiesis, leukemogenesis and certain gene functions [4]. In the context of MPNs, it is well documented that women demonstrate distinct phenotypic profiles compared to men, with higher frequencies of essential thrombocytosis and unique thrombotic and vascular patterns that influence diagnosis and management [5,6,7]. Importantly, sex has been associated with differential symptom burden in MPN patients. For example, female patients report more severe and frequent symptoms across validated assessment tools, despite similar prognostic scores, with abdominal pain and microvascular manifestations (headache, dizziness) being predominantly reported. Interestingly, there were no sex-dependent differences in quality of life [8,9].

The above sex-dependent differences are further compounded in women with myeloproliferative neoplasms as they face distinct reproductive and life-course-specific challenges, including issues related to fertility, pregnancy and menopause [10,11,12]. However, despite this, data for supporting women through these life challenges remains limited, with ongoing sex/gender imbalances in clinical trial participation, including MPNs [13].

Menopause is defined clinically as the permanent cessation of menstruation, diagnosed retrospectively after 12 consecutive months without a menstrual period, and reflects the end of a woman’s natural reproductive lifespan [14]. In the UK, the average age at natural menopause is approximately 51 years, with many women transitioning between 45 and 55 years of age [15]. Menopause constitutes a pivotal phase for women with myeloproliferative neoplasms because it intersects with hormonal, vascular, and hematologic changes that can affect disease course and clinical outcomes [13]. Menopausal transition may also amplify common MPN-related symptoms such as fatigue, sleep disturbance, and vasomotor instability, which can complicate clinical assessment and impact quality of life [9,16]. Furthermore, even if menopause is recognized in women with MPN, there is often a reluctance to start hormone replacement therapy (HRT) due to the potential thrombosis risk, and thus, often treatment can be delayed in this cohort.

In this context, “menopause management” refers not only to the treatment of vasomotor and genitourinary symptoms, but also to the assessment and mitigation of long-term health risks, including cardiovascular disease, thrombosis, osteoporosis, and psychosocial morbidity. For women with MPNs, menopause management must therefore be integrated within disease-specific risk stratification, symptom assessment, and long-term survivorship care. We aim to highlight the clinical challenges and therapeutic considerations to consider for women with MPN, with a proposed algorithm for the management of menopause in this cohort.

## 2. Hormonal Changes and MPNs

Sex hormones influence hematopoiesis, immune regulation, and vascular biology, and estrogen in particular modulates endothelial function, platelet activation, and inflammatory cytokine signaling [3,17,18,19,20,21]. The menopausal transition is characterized by declining estrogen levels and is associated with adverse changes in vascular tone, lipid profiles, insulin sensitivity, and arterial stiffness, contributing to increased cardiovascular risk in women [22,23]. In women with myeloproliferative neoplasms, these menopause-related vascular changes occur in parallel with an established prothrombotic and inflammatory disease milieu, raising the possibility of additive or synergistic effects on thrombosis risk.

MPNs are characterized by chronic inflammation and “thrombo-inflammation,” in which clonal myeloid cells promote endothelial activation and dysfunction, platelet–leukocyte aggregation, neutrophil extracellular trap (NET) formation, and increased tissue factor activity, collectively amplifying thrombotic potential. These pathways may be particularly relevant during menopause, when estrogen withdrawal is associated with reduced endothelial nitric oxide bioavailability and an increase in pro-inflammatory signaling [20,21,22,23]. In addition, mutation-associated vascular risk is central to MPNs; for example, *JAK2*V617F is linked to higher thrombotic risk, potentially through heightened leukocyte activation, altered platelet function, and endothelial perturbation. Thus, menopausal changes may intersect with MPN biology through shared vascular and inflammatory axes rather than through ovarian hormone effects alone [23,24].

Although direct studies examining menopause as a modifier of MPN course are limited, thrombotic events are clearly associated with adverse outcomes in MPNs and may reflect more aggressive disease biology. Barbui et al. demonstrated in a large cohort of patients with polycythemia vera that thrombosis was associated with increased risks of death and disease progression, supporting the concept that thrombosis is not only a complication but also a marker of disease aggressiveness [25]. Moreover, cardiovascular risk factor control is increasingly recognized as a core component of MPN management, and this aligns with menopause care priorities in the general population [14,15,26]. These observations support the pragmatic view that menopause management in women with MPNs should include aggressive modification of cardiovascular risk factors and careful thrombotic risk assessment.

Key gaps in this area include whether menopausal transition independently increases thrombosis risk in women with MPNs beyond established MPN-related risk factors, whether symptom trajectories during menopause correlate with inflammatory markers or clonal burden, and whether menopause influences thresholds for initiating cytoreductive therapy. Similarly, robust data are lacking on how different hormone therapy formulations affect coagulation and thrombotic outcomes, specifically in MPN populations. Prospective, sex-informed studies incorporating symptom measures, biomarkers of inflammation and endothelial activation, and clinical outcomes are needed to clarify these relationships and to support evidence-based guidance for menopause management in MPNs.

## 3. Menopause Symptoms Versus MPN Symptoms: Diagnostic and Clinical Overlap

**Figure 1:**
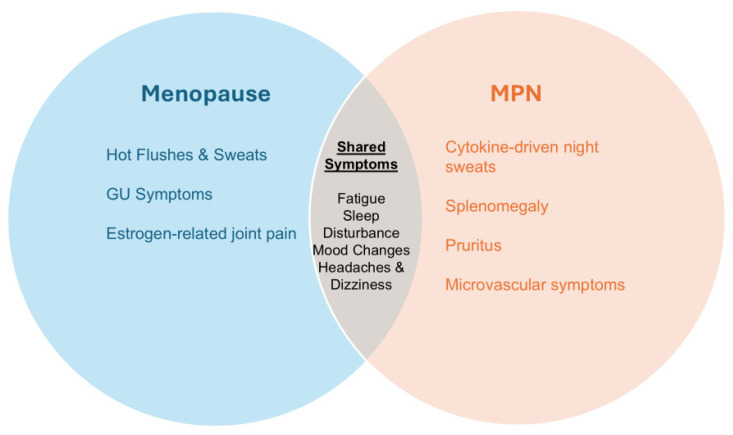
Overlap between Menopausal Symptoms and Myeloproliferative Neoplasm (MPN)-Related Symptoms.

As previously mentioned, menopausal symptoms can be challenging to delineate from MPN, which can impact clinical management due to underdiagnosis of the former or misattribution of the latter (Figure 1).

### 3.1. Vasomotor and Constitutional Symptoms

Hot flushes, night sweats, fatigue and sleep disturbance are hallmark menopausal symptoms, but are also common in MPNs due to cytokine-driven inflammation [27,28,29]. Hot flushes and night sweats can predominate during menopause, with their prevalence being highest in the first year after the final menstrual period. Furthermore, while women may experience these symptoms for less than five years, some may continue to have hot flushes beyond the age of 60 years [30]. In relation to MPN, both hot flushes and night sweats can often be interpreted as disease-related rather than menopausal, thus potentially delaying appropriate symptom management. One way to help distinguish between menopausal and MPN-related vasomotor symptoms is to consider symptom timing and pattern, although this should be viewed as a clinical clue rather than a diagnostic rule. MPN-related night sweats are often persistent, drenching, and associated with other systemic features such as weight loss or splenomegaly, reflecting cytokine-driven inflammation [28]. By contrast, menopausal hot flushes may be more episodic, frequently triggered by environmental or emotional factors, and commonly associated with flushing and palpitations [30,31]. Menopausal symptoms may also cluster during the perimenopausal period and fluctuate over time. Careful longitudinal history-taking and symptom diaries can therefore be helpful in differentiating symptom drivers.

### 3.2. Neurocognitive and Mood Changes

Cognitive impairment (‘brain fog’), fatigue and mood lability are prevalent during menopause, but are also reported by patients with MPNs [32,33]. While neurocognitive changes in MPN are typically related to inflammatory cytokines, the impact of estrogen depletion in menopause has remained underexplored. Recent studies have identified that estrogen may play a critical role in neurocognition via its interaction with estrogen receptors, which are widely expressed in areas of the brain associated with emotion and cognition [34]. Therefore, when estrogen levels decline during menopause, these estrogen receptors become less active and can contribute to the onset of cognitive decline and mood dysregulation experienced by women during this time [35,36].

As mentioned previously, microvascular manifestations (particularly headaches) can predominate in women with MPN, while headaches and migraines can worsen during menopause due to fluctuating estrogen levels [8,37]. This highlights, yet again, the crossover between menopause and MPN in women. However, as discussed earlier, one way to distinguish between these conditions is by identifying when these symptoms are worse—typically, for menopause, headaches are particularly severe in the mornings due to the rapid decline in estrogen levels. By contrast, in MPN, headaches typically worsen during the day due to microvascular blockage by platelets and red cells, with symptoms improving with low-dose aspirin [38].

From a practical perspective, clinicians should maintain a low threshold to screen for depression and anxiety in women with MPNs undergoing menopause, particularly when symptoms persist beyond expected menopausal transition periods, significantly impair daily functioning, or are accompanied by sleep disturbance or social withdrawal. Brief validated tools such as the Patient Health Questionnaire-9 (PHQ-9) and Generalized Anxiety Disorder-7 (GAD-7) can be incorporated into routine visits [39,40]. Moderate-to-severe scores, suicidal ideation, or substantial functional impairment should prompt referral to primary care or mental health services. Recognizing and addressing mood disorders is important, as both menopause and MPN independently increase vulnerability to psychological distress.

### 3.3. Musculoskeletal and Genitourinary Symptoms

Arthralgia and myalgia are commonly reported in menopause, although the causes of joint pains can be difficult to determine, as this time-period coincides with the rising incidence of chronic rheumatic conditions such as osteoarthritis [41]. However, despite this, arthralgia is more prevalent in women going through menopause and is thought to be related to reduced estrogen levels [42]. As mentioned earlier, estrogen has been shown to affect inflammatory signaling as well as cytokine production [21]. When estrogen levels rapidly decline, as observed in menopause, this has been associated with increased levels of reactive oxygen species as well as pro-inflammatory cytokines, which can contribute to arthralgia and myalgia. Importantly, in mouse work, when estrogen receptors have been deleted, this has resulted in cartilage damage and osteophytosis, suggesting estrogens play a protective role in maintaining joint homeostasis [43].

By contrast, musculoskeletal symptoms are rarely associated with MPN, and therefore, it is possible to distinguish this from menopause in women. However, MPN-related treatments, particularly pegylated interferon (Peg IFN), can be associated with arthralgia and myalgia, and therefore it is important to ensure treatment-related toxicity does not overshadow menopause-related symptoms [44,45]. One way to manage this is by identifying when these symptoms are prevalent—in relation to Peg IFN, typically these symptoms develop in the first twenty-four hours after administration and often are associated with flu-like symptoms [44,45]. By contrast, similar to what has been discussed previously, arthralgia and myalgia in relation to menopause are typically worse in the mornings due to the low estrogen levels at this time [42].

As expected, genitourinary (GU) symptoms are common after menopause, although they may also occur in pre- and perimenopausal women [46,47]. Symptoms can include dryness, burning, itching and dyspareunia, as well as urinary tract problems. While one would not expect MPNs to be directly associated with these symptoms, the impact of menopause from a GU perspective can be significant in terms of interpersonal relationships and quality of life, in particular, sexual function [48]. However, it is important to be aware that sexual dysfunction has also been reported in patients with MPNs, particularly in ET and PV, and is thought to be related to microvascular and macrovascular disturbances as well as systemic inflammatory burden [49]. Therefore, in women with both MPN and menopause, genitourinary and sexual symptoms should not be attributed to menopause alone. A combined assessment considering hormonal status, vascular risk, medication effects, and psychosocial factors is often required [50].

## 4. Treatment Considerations for Menopausal Women with MPNs

The management of menopausal symptoms in women with MPNs must balance symptom relief with thrombotic and cardiovascular safety.

Illustrative Case 1: Systemic HRT in a Low-Thrombotic-Risk Patient

A 49-year-old woman with CALR-mutated essential thrombocythemia, no prior thrombotic events, well-controlled platelet counts on pegylated interferon, and no cardiovascular risk factors presents with severe vasomotor symptoms affecting sleep and work performance. After multidisciplinary discussion and shared decision-making, transdermal estrogen combined with micronized progesterone was initiated at the lowest effective dose, with continued low-dose aspirin and close monitoring. Symptoms improved within 8 weeks, with no thrombotic or hematologic complications.

### 4.1. Hormone Replacement Therapy

In the general menopausal population, hormone replacement therapy (HRT) can effectively relieve symptoms of menopause and typically combines estrogen and progesterone to mimic ovarian hormones [14,15]. However, historically, the use of HRT in women with MPN has been controversial due to previous studies demonstrating an increased risk of venous thromboembolism (VTE) and arterial events [51]. Regarding the latter (particularly ischemic strokes), though more recent studies have suggested this risk is primarily in older women (age over 60 years) who had initiated HRT over 10 years after the onset of menopause [52]. In relation to venous thrombosis, the Women’s Health Initiative (WHI) trial demonstrated increased risk of VTE, particularly within the first two years of starting HRT [53]. A more recent study led by Johansson et al. (2024) confirmed these findings using a large-scale Swedish national registry [54]. In this study, over 70,000 women initiated HRT, with 24,089 having an event recorded during follow-up. These events included ischemic heart disease, ischemic stroke and venous thromboembolism, with the latter being the most common, affecting over one-third of women [54]. Notably, there was a strong correlation with the type of HRT used and rates of thrombotic events, with both oral and combined (estrogen and progesterone) preparations being associated with increased risk of ischemic heart disease as well as venous thromboembolic events. By contrast, transdermal and estrogen-only preparations were associated with a lower risk of embolic events [54].

In women with MPN, baseline thrombotic risk is already elevated due to disease-related factors including clonal hematopoiesis, inflammation and endothelial activation [24]. Venous thromboembolic events in particular are more frequent in women, which is often compounded by additional risk factors including exogenous hormone therapy, pregnancy and hereditary thrombophilias. Furthermore, the presence of *JAK2*V617F mutation in combination with these risk factors has been associated with an increased relative risk of VTE in women with MPN [55]. Therefore, this necessitates a more cautious and individualized approach. However, direct evidence on systemic HRT in MPN populations is limited, and recommendations are therefore extrapolated from general population data combined with MPN assessment.

Based on the above, when considering HRT in women with MPN, the following factors need to be taken into consideration:

(i) Thrombotic history: prior arterial or venous thrombosis substantially increases risk
(ii) Driver mutation status: *JAK2*V617F is associated with higher thrombosis risk
(iii) Cardiovascular risk factors
(iv) Disease control: women with poorly controlled hematocrit or blood counts would be at higher risk of thrombotic events

In women with a history of thrombosis, systemic HRT is generally avoided or considered only after multidisciplinary discussion. If it is used, it should be in the context of optimal MPN management, including cytoreduction and antiplatelet/anticoagulant therapy [55,56]. If systemic HRT is used, micronized progesterone or dydrogesterone is often favored because they have been associated with a lower risk of both arterial and venous thrombotic events, although MPN-specific data are lacking [54]. Women with MPNs started on systemic HRT should have a structured follow-up, including symptom response review at 3 months, blood pressure monitoring, education regarding VTE symptoms, assessment of cardiovascular risk and ongoing review of MPN disease control.

Transdermal estrogen is generally preferred over oral preparations of HRT in women with MPN, due to the reduced impact on coagulation pathways and thus lower thrombotic risk [54]. This is highlighted by Case 1 above, where a patient with *CALR-*mutated essential thrombocythemia was commenced on transdermal HRT with relief of symptoms within a few months. Furthermore, as with Case 1, HRT should be prescribed with the lowest effective dose, especially as treatment may be required for around five years for hot flushes. In those women who experience premature menopause, HRT should continue until they are in their early 50 s, not only to treat symptoms but also to reduce the risk of osteoporosis [14,57].

At present, there is limited evidence on whether it is better to taper down or to stop HRT abruptly after a specified period of time. Therefore, the current recommendation is to continue HRT for as long as there is clinical benefit [13,58]. However, as mentioned previously, these guidelines are based on the general population. In women with MPN, we would emphasize that the prescribing responsibility should ideally be shared between a menopause specialist and the hematologist, with clear documentation of risk-benefit discussions and patient preference. Furthermore, concurrent use of antiplatelet/anticoagulant therapy does not eliminate HRT-associated thrombotic risk. Therefore, decisions on HRT as well as treatment duration should be individualized, using the lowest effective dose for the shortest time period consistent with symptom control, while recognizing some women may require longer therapy [57,58].

Illustrative Case 2: Local Vaginal Estrogen in High-Risk Disease

A 62-year-old woman with post-ET myelofibrosis and prior splanchnic vein thrombosis reports significant dyspareunia and recurrent urinary symptoms. Low-dose vaginal estradiol tablets were prescribed after counseling regarding minimal systemic absorption. Symptoms improved without thrombotic recurrence.

### 4.2. Local Estrogen Therapy

Genitourinary syndrome of menopause (GSM) can significantly impair quality of life and sexual function. Low-dose vaginal estrogen preparations are highly effective for GSM and generally result in minimal systemic estrogen exposure compared with systemic HRT [46,47]. Systemic absorption varies by preparation, whereby vaginal tablets and rings typically have the lowest systemic levels, while creams are slightly higher but still have low systemic absorption when used at recommended doses. Furthermore, serum estradiol levels usually remain within postmenopausal ranges with the low-dose regimens, and can help reduce vaginal dryness [13,47].

As systemic exposure is low, local vaginal estrogen can be considered in women with higher thrombotic risk, as highlighted by Case 2 above, although data specific to MPNs are limited. In women with very high thrombotic risk, or those who prefer to avoid hormones, non-hormonal options are available for GSM, including vaginal moisturizers and lubricants, pelvic floor therapy and regular vaginal dilators to maintain tissue health [46,47].

## 5. Alternatives to Hormone Replacement Therapy

For women in whom systemic HRT is contraindicated, declined, or considered high risk, non-hormonal options can play a central role in symptom management, as highlighted by Illustrative Case 3. In women with MPNs, these approaches are often particularly relevant given baseline thrombotic risk.

Illustrative Case 3: Menopause Management in a Woman With Prior Thrombosis

A 56-year-old woman with *JAK2*-positive polycythemia vera and a history of unprovoked deep vein thrombosis on long-term apixaban presents with severe hot flushes and night sweats. Systemic HRT was avoided due to high thrombotic risk. Non-hormonal therapy with venlafaxine was initiated alongside lifestyle measures, resulting in partial symptom improvement.

### 5.1. Pharmacological Options

#### 5.1.1. Selective Serotonin Reuptake Inhibitors (SSRIs)/Selective Norepinephrine Reuptake Inhibitors (SNRIs)

Both SSRIs and SNRIs have been shown to be effective at relieving vasomotor symptoms of menopause. A 2015 systematic review of 18 randomized controlled trials evaluated the effectiveness of these treatments in women with menopause. Overall, the SSRI Paroxetine demonstrated the greatest overall reduction in hot flushes compared to placebo, while the SNRI Venlafaxine had the fastest onset for symptom relief [59]. The North American Menopause Society published an evidence-based position paper the same year, with particular focus on non-hormonal options for treatment of vasomotor symptoms. Overall, it was concluded that both SSRIs and SNRIs provided significant symptom relief, although the former had fewer initial adverse effects. It is important to note that this position paper also highlighted that SSRIs could impair tamoxifen metabolism, and therefore SNRIs (Venlafaxine preferably) should be used in women with a history of breast cancer [60].

Typical starting doses for these drugs are summarized below:

- Paroxetine: 7.5–10 mg daily
- Escitalopram: 5–10 mg daily
- Venlafaxine: 37.5 mg daily (titrate to 75 mg if needed)

Common adverse effects associated with SSRIs/SNRIs include nausea, dry mouth, sleep disturbance and sexual dysfunction [59,60]. In women with MPN, these drugs do offer an alternative option for women with significant vasomotor symptoms who cannot use HRT, including those with prior thrombosis or high cardiovascular risk. These agents may also be beneficial in those women with associated anxiety/depression. However, it is important to be cautious in those on anticoagulant therapy (especially direct oral anticoagulants; DOACs) due to potential drug–drug interactions with SSRIs/SNRIs [61].

#### 5.1.2. Gabapentin

Gabapentin, which is usually used as an anticonvulsant, has been shown to alleviate hot flushes. In one study, this drug was compared to estrogen in women with menopause over one year. Although the sample size was small, there was a significant decrease in both severity and frequency of hot flushes, even in women taking a lower dose of Gabapentin at 100 mg daily. Furthermore, this drug was well tolerated with few adverse effects and no treatment discontinuation [62]. Previous studies had used much higher doses of Gabapentin, ranging from 300 mg to 2400 mg per day for 4–12 weeks; however, the higher doses were associated with adverse effects, including headache and dizziness [63]. In general, it is advised to start at a lower dose of Gabapentin (typically 100–300 mg at night), with gradual titration to 900 mg/day in divided doses according to tolerability. Adverse effects can include drowsiness, dizziness and fatigue—therefore, it is important to closely monitor their response to this drug and adjust dosing accordingly [63]. Gabapentin can be particularly useful in women with MPN with significant nocturnal symptoms or sleep disturbance, and therefore, they should ideally take this medication at night for maximal benefit [64].

#### 5.1.3. Clonidine

Clonidine, an alpha2-adrenergic agonist, has been shown to reduce hot flushes by decreasing noradrenergic activity in the blood vessels [65]. Typical starting doses range between 25–50 micrograms twice daily. However, due to its mechanism of action, Clonidine is associated with significant cardiovascular adverse effects, including bradycardia and orthostatic hypotension, as well as dry mouth and constipation [66]. In women with MPN, Clonidine may be considered if other options are ineffective or contraindicated, but its toxicity profile does limit tolerability. It should also be used with caution in women with known cardiovascular disease, and avoided altogether if there are significant cardiovascular comorbidities.

### 5.2. Non-Pharmacological Interventions

Non-pharmacologic strategies are low-risk and may provide meaningful symptom relief, particularly when combined with other therapies [15]. Cognitive Behavioral Therapy (CBT) has been shown to improve coping with vasomotor symptoms and insomnia [67]. This can be particularly important for women with MPN, as insomnia can be a predominant symptom and can severely impact quality of life [9,68]. Regular physical activity has been associated with improved mood, sleep quality, and overall well-being, although its impact on hot flushes has been modest. Weight management has also been shown to improve menopause-related symptoms, with weight loss being associated with reduced vasomotor symptom severity [14,26]. Sleep optimization is also important, with consistent sleep schedules and sleep hygiene strategies shown to mitigate fatigue and insomnia. These lifestyle measures are particularly relevant in women with MPNs, as both fatigue and sleep disturbance are common contributors to reduced quality of life [9,68].

### 5.3. Bone Health Management

Menopause significantly increases the risk of osteoporosis in women due to falling estrogen levels, causing accelerated bone loss, with women losing up to 20% of bone density in the first few years after menopause [15]. Therefore, baseline bone mineral density assessment should be considered if any of the following [69]:

- Women age ≥ 65 years
- Postmenopausal women aged < 65 years with risk factors
- Women with premature menopause
- History of fragility fracture
- Body Mass Index less than 22 kg/m^2^
- Prolonged corticosteroid exposure
- Chronic inflammatory disease (e.g., Rheumatoid Arthritis)
- Prolonged immobility

Given the chronic inflammatory state of MPNs and potential treatment-related effects, earlier assessment of bone mineral density may be considered in selected patients.

There has been emerging data suggesting that MPNs are also associated with increased rates of both osteoporosis and osteoporotic fractures, with contributing risk factors including age > 70 years, female sex and presence of comorbidities [70]. Some MPN therapies and advanced disease states may also indirectly affect bone health through systemic inflammation and reduced mobility [70]. Therefore, high-risk women with MPN should receive standard osteoporosis prevention and treatment strategies, including:

- Calcium and Vitamin D optimization—the latter is particularly relevant to women with MPNs, as both PV and PMF in particular have been associated with increased rates of vitamin D deficiency [71]
- Weight-bearing exercises
- Bisphosphonates when indicated [72]

HRT can also help to mitigate this risk and should be offered to women, based on initial risk assessment as discussed earlier.

## 6. Psychosocial and Emotional Well-Being

From a practical perspective, clinicians should maintain a low threshold to screen for psychological distress in women with MPNs undergoing menopause, particularly when symptoms persist, worsen, or impair daily functioning. Brief validated tools such as the Patient Health Questionnaire-9 (PHQ-9) for depression and the Generalized Anxiety Disorder-7 (GAD-7) for anxiety can be incorporated into routine visits [39,40]. Clinically significant scores, suicidal ideation, or marked functional impairment should prompt referral to primary care or mental health services.

Referral to psychology or counseling services may be particularly helpful for coping with chronic illness and menopausal transition. In addition, women reporting genitourinary or sexual concerns should be offered referral to menopause or sexual health specialists, as these symptoms are common yet frequently under-discussed [73]. A multidisciplinary, supportive approach can substantially improve quality of life in this population. Integrating psychosocial screening into routine care and facilitating access to mental health support, sexual health specialists and peer support networks is essential to the holistic management of these women.

## 7. Collaborative and Multidisciplinary Care Approach

As highlighted by this review article, menopause management in MPNs is complex and therefore a multidisciplinary model is critical. Optimal care involves collaboration between hematologists, gynecologists or menopause specialists, primary care physicians, cardiologists and mental health professionals. Importantly, shared decision-making and individualized risk assessment should guide therapy selection when managing menopause, and it is important to regularly assess patients.

Menopause may also indirectly influence thresholds for MPN-directed therapy. Worsening fatigue, vasomotor symptoms, sleep disturbance, or mood changes during the menopausal transition may amplify overall symptom burden and affect patient-reported outcomes, potentially prompting earlier initiation or escalation of cytoreductive therapy. Conversely, concerns regarding cardiovascular and thrombotic risk during menopause may influence treatment selection, particularly in women with additional risk factors. Bone health considerations may also impact therapeutic decision-making, as cytoreductive agents and disease-related inflammation could exacerbate postmenopausal bone loss. These factors underscore the importance of holistic assessment when determining MPN treatment strategies in menopausal women.

**Figure 2:**
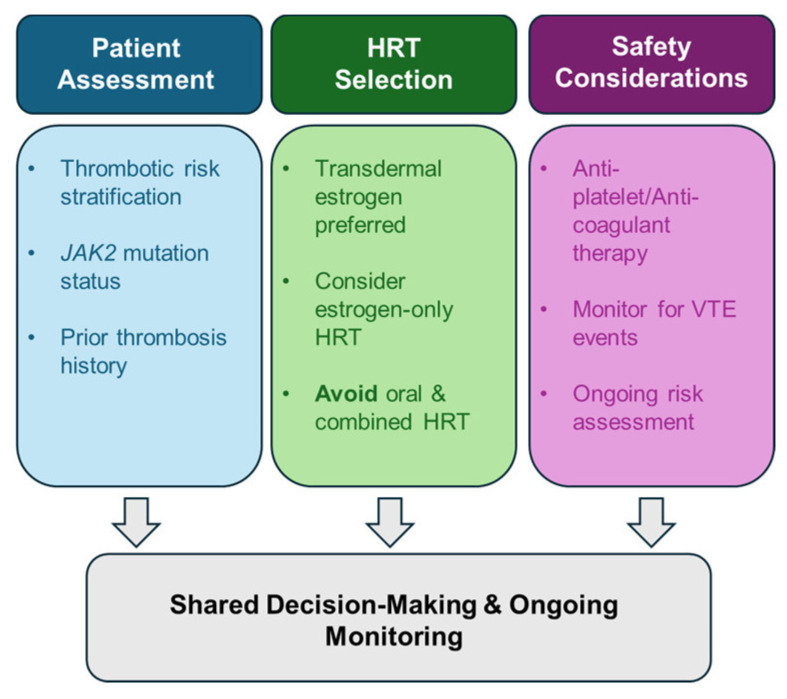
Hormone Therapy Decision Framework in Women with Myeloproliferative Neoplasms.

**Figure 3:**
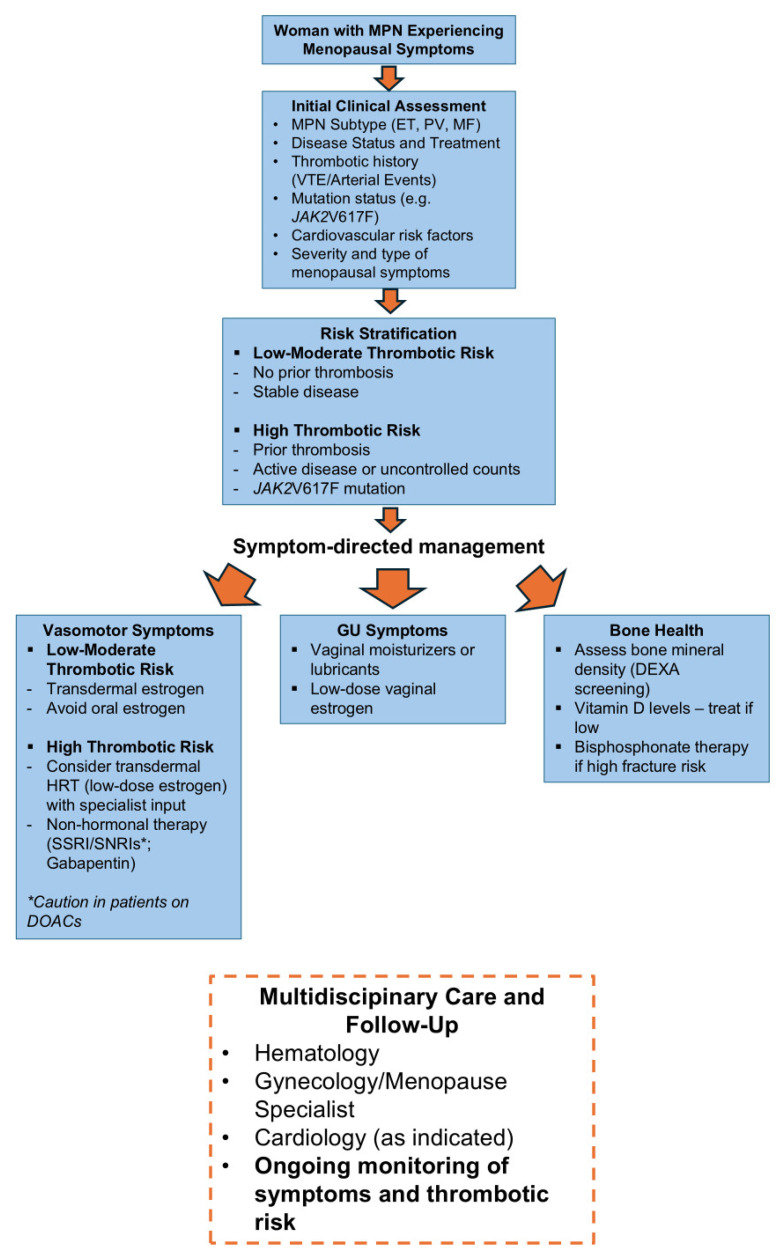
Proposed Algorithm for the Management of Menopause in Women with Myeloproliferative Neoplasms.

Given the absence of disease-specific guidelines, we have developed a pragmatic clinical algorithm for the management of menopausal symptoms in women with MPNs, as shown in Figure 2 and Figure 3.

## 8. Conclusions

Menopause represents a critical and under-recognized phase in the care of women with myeloproliferative neoplasms. Hormonal changes during menopause intersect with MPN-related inflammation, thrombotic risk, symptom burden, and long-term comorbidities, creating unique clinical challenges. Overlapping symptomatology may complicate diagnosis and delay appropriate management, while concerns regarding thrombosis often limit access to effective therapies such as hormone replacement therapy.

Although systemic HRT may be appropriate for carefully selected women with low thrombotic risk, non-hormonal strategies and supportive interventions remain central to management in this population. Given the lack of disease-specific guidance, individualized risk assessment, shared decision-making, and multidisciplinary collaboration are essential. Importantly, current recommendations are largely extrapolated from the general menopausal population, highlighting a critical gap in evidence specific to women with MPNs. Prospective studies evaluating the safety of hormone therapy, the impact of menopause on disease trajectory, and patient-reported outcomes are urgently needed. Development of disease-specific guidelines will be essential to optimize menopause care within MPN survivorship.

**Top 5 Clinical Takeaways**

(i) **Symptom overlap is common:** Menopausal symptoms frequently overlap with MPN-related manifestations, requiring careful longitudinal assessment to guide management.
(ii) **Thrombotic risk is central:** Decisions regarding systemic hormone replacement therapy must be individualized and guided by MPN subtype, thrombotic history, and overall cardiovascular risk.
(iii) **Non-hormonal strategies are essential:** Pharmacologic and non-pharmacologic alternatives to HRT are key components of care, particularly in women at higher thrombotic risk.
(iv) **Holistic care improves outcomes:** Attention to bone health, cardiovascular risk, sexual health, and psychosocial well-being is critical during menopause in women with MPNs.
(v) **Multidisciplinary management is recommended:** Collaborative care between hematology, gynecology, and primary care supports safe, patient-centered menopause management.

## Data Availability

There is no new data created from this article.

## Abbreviations

The following abbreviations are used in this manuscript:

DOAC	Direct Oral Anticoagulant
ET	Essential Thrombocythemia
GU	Genito-Urinary
HRT	Hormone replacement therapy
IFN	Interferon
MPN	Myeloproliferative Neoplasm
MF	Myelofibrosis
Peg	Pegylated
PV	Polycythemia vera
SNRI	Selective norepinephrine reuptake inhibitor
SSRI	Selective serotonin reuptake inhibitor
VTE	Venous Thromboembolism
WHI	Women’s Health Initiative
